# Resistance Status to the Insecticides Temephos, Deltamethrin, and Diflubenzuron in Brazilian *Aedes aegypti* Populations

**DOI:** 10.1155/2016/8603263

**Published:** 2016-06-21

**Authors:** Diogo Fernandes Bellinato, Priscila Fernandes Viana-Medeiros, Simone Costa Araújo, Ademir J. Martins, José Bento Pereira Lima, Denise Valle

**Affiliations:** ^1^Laboratório de Biologia Molecular de Flavivírus, Instituto Oswaldo Cruz, Fiocruz, Rio de Janeiro, RJ, Brazil; ^2^Laboratório de Fisiologia e Controle de Artrópodes Vetores, Instituto Oswaldo Cruz, Fiocruz, Rio de Janeiro, RJ, Brazil; ^3^Instituto de Biologia do Exército, Rio de Janeiro, RJ, Brazil; ^4^Gerência de Controle de Zoonoses, Secretaria Municipal de Saúde, Belo Horizonte, MG, Brazil; ^5^Instituto Nacional de Ciência e Tecnologia em Entomologia Molecular (INCT-EM), Rio de Janeiro, RJ, Brazil

## Abstract

Insecticides are still largely applied in public health to control disease vectors. In Brazil, organophosphates (OP) and pyrethroids (PY) are used against* Aedes aegypti* for years. Since 2009 Insect Growth Regulators (IGR) are also employed in the control of larvae. We quantified resistance to temephos (OP), deltamethrin (PY), and diflubenzuron (IGR) of* A. aegypti* samples from 12 municipalities distributed throughout the country, collected between 2010 and 2012. High levels of resistance to neurotoxic insecticides were detected in almost all populations: RR_95_ to temephos varied between 4.0 and 27.1; the lowest RR_95_ to deltamethrin was 13.1, and values higher than 70.0 were found. In contrast, all samples were susceptible to diflubenzuron (RR_95_ < 2.3). Biochemical tests performed with larvae and adults discarded the participation of acetylcholinesterase, the OP target, and confirmed involvement of the detoxifying enzymes esterases, mixed function oxidases, and glutathione-*S*-transferases. The results obtained were discussed taking into account the public chemical control component and the increase in the domestic use of insecticides during dengue epidemic seasons in the evaluated municipalities.

## 1. Introduction

Currently dengue is spreading worldwide, placing at risk around 40% of the global population [1]. To date, no specific drugs are available and dengue treatment is restricted to supportive care. Although several candidate vaccines, directed against the four dengue serotypes, are presently submitted to human clinical trials, or even licensed for commercialization, none of them attains high protection levels [2]. The major dengue vector is* Aedes aegypti* (Diptera: Linnaeus, 1762), a highly anthropophilic and synanthropic mosquito, distributed throughout tropical and subtropical areas of the world [3–5], mainly between latitudes 35°N and 35°S [6, 7]. In addition, the recent chikungunya and Zika virus dispersion throughout the globe is also primarily attributed to* A. aegypti* [8].

Actions against dengue are mostly focused on the reduction of mosquito densities, and vector control can be accomplished through mechanical, biological, and chemical approaches. Mechanical control is based on the elimination or on the adequate protection of potential breeding sites; biological control makes use of larvae predators, such as small fishes, or formulations with entomopathogenic bacteria, like* Bacillus thuringiensis var. israelensis* (*Bti*); chemical control consists in the use of insecticides against larvae or adults of the vector mosquito [6, 7, 9].

Insecticides, still largely utilized by a number of vector control programs, belong to four main classes; all of them are neurotoxic compounds: carbamates (CA), organochlorates (OC), organophosphates (OP), and pyrethroids (PY) [10]. Nowadays, PY and OP are the most used. Recently two additional classes became available, the spinosyns, modulators of acetylcholine receptors [11], and the Insect Growth Regulators (IGR), a group that includes the chitin synthesis inhibitors (CSI) [12]. It should be noted that the Brazilian Dengue Control Program (PNCD) only employs insecticides that are recommended by the World Health Organization Pesticide Evaluation Scheme (WHOPES) for use in potable water or properly approved for space spraying applications [13, 14].

The intensive and prolonged use of insecticides can select resistant specimens in the natural vector populations, decreasing the frequency of susceptible individuals and reducing variability of field populations [5]. Insecticide resistance can derive from different mechanisms, the main ones being modifications in the target sites and higher ability to detoxify xenobiotic compounds; the former mechanism is known as target site resistance and the other as metabolic resistance [5, 6].

The voltage gated sodium channel (Na_V_) is the target site of pyrethroids; these insecticides keep Na_V_ in its opened conformation, resulting in repetitive pulses. Na_V_ substitutions that affect its susceptibility to PY are known as knockdown resistant ones (kdr) [15]. Such mutations have been reported in* A. aegypti* populations from several countries worldwide [16–18]. In Brazil two major kdr Na_V_ alleles related to PY resistance are spreading and increasing in frequency. A clear regional distribution pattern is observed with Na_V_^R1^ (mutant at position 1534 of the channel protein) present throughout the country while Na_V_^R2^ (mutant at both 1534 and 1016 positions) is more frequent in central and southeastern municipalities [19, 20].

The target site of OP insecticides is acetylcholinesterase (AChE), an enzyme that hydrolyzes acetylcholine molecules; as a consequence, this neurotransmitter persists in the synaptic cleft, resulting in the exacerbation of nerve impulse transmission [21, 22]. To our knowledge there are no confirmed evidences of AChE alterations related to OP resistance in field* A. aegypti* populations.

The main detoxifying enzyme classes participating in the xenobiotic metabolizing processes are the Phase I mixed function oxidases (MFO) and esterases (EST) that trigger chemical modifications in the substrates and the Phase II conjugating enzymes, glutathione-*S*-transferases (GST) [21]. Each of these enzyme families is composed of several molecular entities, bearing distinct levels of specificity [5]. In general, evaluations of* A. aegypti* detoxifying mechanisms worldwide associate ESTs and OP resistance as well as GST and MFO alterations with PY resistance [23–26]. However such relations are not always straightforward due to the variability of enzymes participating in the insecticides detoxification and to the resistance multifactorial character [5, 10].

In Brazil, during more than three decades, only temephos was employed in the control of* A. aegypti* larvae. Resistance to this OP was originally detected at the end of the years 1990, and registers of the dissemination of this phenomenon persist up to the present [23, 24, 27–30]. Since 2009 temephos is being substituted by IGR in the country and a strategy of larvicide rotation, each 3-4 years, is attempted. Development of resistance was also verified for PY shortly after its use has been implemented for the control of adults, since 2000 [23, 31, 32].

**Figure 1:**
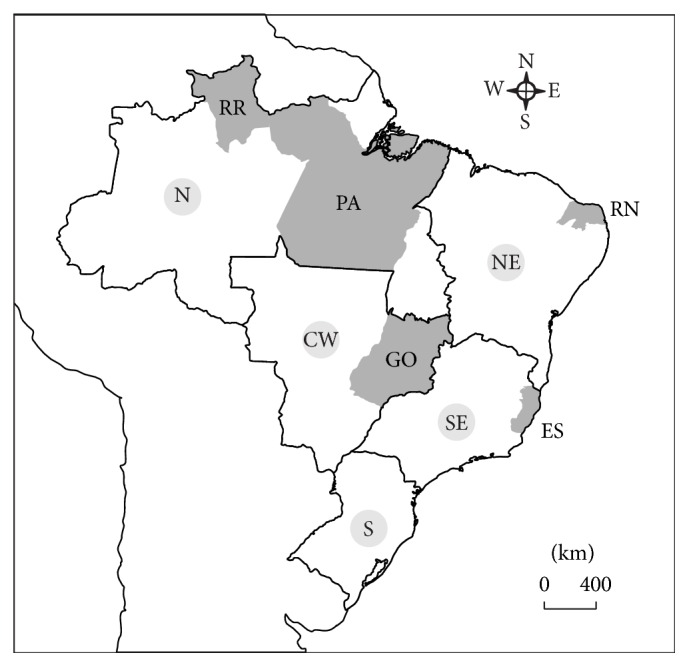
Brazilian map with the states used in the study in grey. RR: Roraima; PA: Pará; RN: Rio Grande do Norte; ES: Espírito Santo; and GO: Goiás. The continuous lines indicate the different regions of the country. N: north; NE: northeast; SE: southeast; S: south; and CW: central-west.

The insecticide susceptibility profiles of several Brazilian field* A. aegypti* populations are presented (Figure 1). Resistance to compounds employed by the PNCD was investigated, in order to collaborate with the elucidation of both the resistance dynamics and the potential related mechanisms. Chemical insecticides are still a relevant control tool employed by public managers against dengue vectors. In addition, in general, dengue epidemic periods are related to a significant increase in the domestic use of insecticides, mainly adulticides. This collective behavior has the potential to contribute to a rapid increase in resistance levels, and it has already been detected in Brazil against pyrethroid compounds [33]. Taking these aspects into account, the results obtained were evaluated in the scope of several parameters, such as the Ministry of Health (MoH) supply of insecticides to the Brazilian States, the local historic of dengue outbreaks, and the frequency of kdr mutations, majorly responsible for PY resistance in the country.

## 2. Materials and Methods

### 2.1. Data on Insecticide Distribution and Dengue Cases

**Figure 2:**
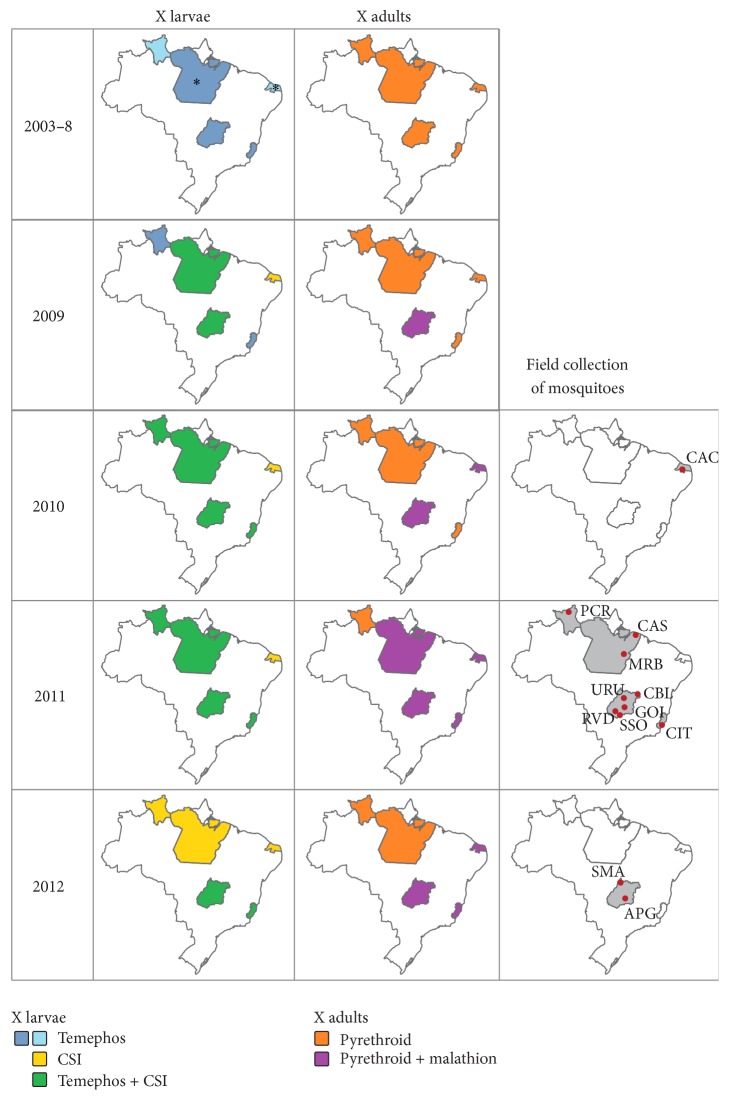
Historic of insecticide distribution, between 2003 and 2012, by the Brazilian MoH to the States where mosquitoes samples were collected, in the indicated years. The municipalities evaluated are highlighted with red dots in the panels at right. During 2003–2008, two states, shown in lighter blue in the upper map at left, did not receive temephos continuously: at Roraima (RR) temephos supply started at 2005; at Rio Grande do Norte (RN) it ended at 2004. (*∗*) States that also received CSI (diflubenzuron or novaluron) during 2003–2008 (RN at 2004 and PA at 2008). Compounds against adults: all pyrethroids distributed by the MoH were considered, and not only deltamethrin (see text for details). Municipalities—APG: Aparecida de Goiânia; CAC: Caicó; CAS: Castanhal; CBL: Campos Belos; CIT: Cachoeiro de Itapemirim; GOI: Goiânia; MRB: Marabá; PCR: Pacaraima; RVD: Rio Verde; SMA: São Miguel do Araguaia; SSO: São Simão; and URU: Uruaçu.

The Brazilian MoH coordinates the distribution of insecticides used in public health to all states and to all disease vector control programs. Insecticides are stored in a warehouse of Rio de Janeiro State Health Secretariat, in charge of stock control and supply of the products to the different States. We got MoH authorization to access these data, sorted by year, since 2003.  Figure 2 illustrates the insecticides employed by PNCD from 2003 until 2012, the latter corresponding to the year of collection of the last samples in the field.

Dengue incidence rates were based on the historical series of cases available at the MoH website for each municipality [34] and on the 2010 population census data conducted by the Brazilian Institute of Geography and Statistics [35].

### 2.2. Mosquitoes

Natural* A. aegypti* populations were collected between 2010 and 2012, in 12 municipalities belonging to a total of five States (Figures 1 and 2): Roraima (RR) and Pará (PA) at the north, Rio Grande do Norte (RN) at the northeast, Espírito Santo (ES), at the southeast, and Goiás (GO) at the central-west region. In all cases, sampling of vector eggs was performed with ovitraps according to MoReNAa (the Brazilian* A. aegypti* insecticide resistance monitoring network) guidelines, as described elsewhere [28, 36]. Depending on the number of buildings in each municipality, 150 to 300 ovitraps were installed during 5–7 days, representing the whole area.

Rockefeller mosquitoes (Rock), a reference strain of insecticide susceptibility [37], were employed as control in all bioassays and also in the biochemical and molecular analysis.

### 2.3. Mosquitoes Rearing

Eggs derived from field populations were allowed to hatch for two days in plastic cups containing 2.5 L of dechlorinated water and a small amount of cat food (Friskies®, Purina, São Paulo, SP). Pools of 1,000 larvae were then transferred to transparent plastic trays (33 × 24 × 8 cm) filled with 1.0 L of water and fed with 0.5 g of cat food every three days. The resulting pupae were transferred to cartoon cages (18 × 17 cm) and the* A. aegypti* emerging female and male adults were separated from other mosquito species, scored and reared in cages in order to proceed to blood feeding and egg laying. Adult females were fed on xylazine and ketamine-anaesthetized guinea pigs [38] for 30 minutes; oviposition cups were placed inside the cages three days later. Achievement of F1 and F2 generations in the laboratory was performed essentially as described elsewhere [28]. The whole procedure took place at 26 ± 1°C and 80 ± 10% relative humidity.

### 2.4. Larval Bioassays

In order to maximize synchronous development, egg hatching was induced during one hour in dechlorinated water. Afterwards, groups of 1,000 larvae were reared in plastic trays, as described above, until the third instar (L3).

Dose response bioassays with temephos (Pestanal®, Sigma-Aldrich) were performed with 10 different concentrations of the OP, designed to kill between 10 and 95% of each population. Four 100 mL replicas were employed per concentration and 20 L3 larvae per replica. Mortality was registered after 24 hours of exposure [29, 39, 40].

For the CSI diflubenzuron (Sigma-Aldrich), each dose response bioassay employed eight insecticide concentrations, also designed to be effective between 10 and 95%. Four 150 mL replicas per concentration and 10 L3 larvae per replica were employed. Both the bioassay methodology and the evaluation criteria were adapted from previous work [41, 42]. In this case, records were made each other day. Replicas were covered with a nylon mesh in order to avoid escaping of adults. The assay was considered terminated when all the specimens from the control group, nonexposed to the CSI, emerged as adults.

Two internal controls were placed at every bioassay: (a) Rockefeller larvae exposed to two different insecticide concentrations, the ED_99_ (effective dose) and half of it, and (b) field specimens kept with the solvent, in the same amount used for the experimental samples.

### 2.5. Adult Bioassays

Female adults were submitted to dose response bioassays to quantify resistance to the pyrethroid deltamethrin (Sigma-Aldrich) following the World Health Organization [43] methodology of impregnated papers, with some modifications [40, 44]. Assays employed 10 deltamethrin concentrations, ideally killing between 10 and 95% of each mosquito population. In all cases three replicas with 15–20 non-blood-fed females, 1–5 day-old, were used. After exposure to the pyrethroid during one hour, mosquitoes were recovered for 24 hours in insecticide-free compartments, when mortality was recorded. Adult bioassay controls followed the same rational employed for larvae ones: Rockefeller specimens exposed to two different deltamethrin concentrations and field derived adults exposed to the solvent.

### 2.6. Biochemical Assays

The potential mechanisms involved with resistance were evaluated through biochemical assays that quantified the activity of several classes of enzymes according to WHO and CDC procedures [23, 45, 46]. Two Phase I enzyme classes were evaluated, MFO and EST. While MFO was indirectly measured, three substrates were employed for EST: *α*- and *β*-naphtyl and *ρ*-nitrophenyl acetates, accounting, respectively, for activities named *α*-EST, *β*-EST, and *ρ*NPA-EST. The Phase II GST and the OP target site AChE were also evaluated. For AChE, both total activity and activity inhibited by the carbamate propoxur were assayed. According to WHO criterion [47], AChE inhibition higher than 70% points to an activity compatible with insecticide susceptibility. Dosage of total proteins was done with the Bio-Rad protein assay/dye reagent concentrate (500-0006), and the results were used to calculate enzymatic specific activities.

Tests with each population employed approximately 90 individual non-blood-fed young females (up to 24 hours after adult emergence) and 90 late L3-early L4 larvae. All specimens were stored at −80°C until use.

Enzyme activities were classified essentially according to what was established previously [23]: after calculating the Rockefeller 99 percentile, the rate of specimens above this value was estimated for each enzyme and population. Activities were classified as unaltered, altered, or highly altered if this rate was, respectively, below 15, between 15 and 50, or above 50%.

### 2.7. Molecular Assays

The results of kdr genotyping were previously published [20] and herein explored in parallel with the bioassays. Briefly, the genotyping was conducted with a customized real-time PCR TaqMan SNP Genotyping Assay (Thermo Fisher), for Val1016Ile (AHS1DL6) and Phe1534Cys (AHUADFA). In general 30 individual adult males preferentially from the parental generation were used in two independent reactions, one for each Na_V_ kdr SNP (1016 and 1534). The allelic and genotypic frequencies of each population were calculated based on variations at both positions, assuming that they are under linkage disequilibrium, which resulted in the alleles Na_V_^S^ (1016 Val^+^ + 1534 Phe^+^), Na_V_^R1^ (1016 Val^+^ + 1534 Cys^kdr^), and Na_V_^R2^ (1016 Ile^kdr^ + 1534 Cys^kdr^) [20].

### 2.8. Interpretation of Results

Results of bioassays for each population and every active compound derived from three or four tests performed in different days. The lethal concentrations (LC) in the case of neurotoxic insecticides or the concentrations inhibiting adult emergence (EI), when the IGR was considered, were calculated using probit analyses [48] (Polo-PC, LeOra Software, Berkeley, CA). Resistance ratios (RR_50_, RR_95_) were acquired dividing the results obtained for each population by the equivalent Rockefeller's values. For all insecticides, the resistance status of mosquito populations was classified according to the criterion utilized in the country to temephos evaluation. This criterion, recommended by PNCD, considers that populations with RR_95_ above 3.0 are resistant [23, 49] (see Section 4).

## 3. Results

### 3.1. Insecticides Employed against* Aedes aegypti* in the Field

Figure 2 exhibits the recent history of insecticides distributed by the Brazilian MoH to the States where field collection of* A. aegypti* populations took place. All larvicides evaluated in the present work are depicted. Beyond these products, Bti was also employed in the field (not shown), during most of the period between 2003 and 2009, except for the central-west State of Goiás. For adulticides, besides the organophosphate malathion, deltamethrin was the pyrethroid elected against* A. aegypti*. However, several PY compounds were also used in the scope of the control of other vectors. Therefore, Figure 2 depicts all pyrethroids distributed for this purpose by the MoH, since these products can interfere with* A. aegypti* populations' susceptibility status. It should also be taken into account that the uncontrolled domestic use of pyrethroids plays an important role in the dissemination of insecticide resistance [32]. However, information regarding domestic use is very difficult to obtain.

Up to 2011, the larvicide temephos was continuously distributed to the states evaluated, with two exceptions: temephos supply to RR started only in 2005 and to RN it lasted until 2004 (Figure 2, light blue states in the 2003–8 line). In this latter state, control of larvae employed Bti between 2005 and 2008. Since 2009-2010 the CSI diflubenzuron was introduced in the* A. aegypti* larvae control, in addition to the organophosphate temephos in all states. The exception was RN where, as mentioned above, temephos had been previously discontinued; in this case, the CSI remained the sole larvicide adopted in* A. aegypti* control from 2009 on.

Control of adult mosquitoes was performed exclusively with pyrethroids between 2003 and 2008. Since 2009, the organophosphate malathion was gradually introduced. At 2011, all the states received both compounds, except RR that employed exclusively PY. At 2012, malathion was not distributed to the state of Pará.

### 3.2. Dengue Incidence in the Evaluated Municipalities 

**Figure 3:**
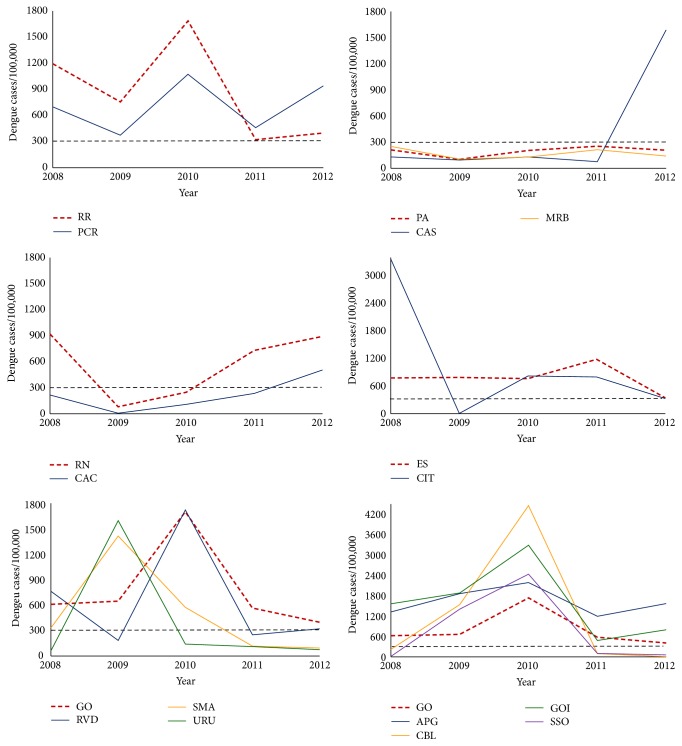
Incidence of reported dengue cases in the municipalities evaluated from 2008 to 2012 (values refer to the rate of notified cases per 100,000 inhabitants). In each panel, the thick red line refers to the dengue incidence in the corresponding State. The dashed line indicates the point above which dengue incidence is considered high. Note that in some panels it was necessary to change the* y*-scale in order to include all values.

Figure 3 shows the incidence of dengue reported cases for all municipalities evaluated here. The aim in this case was to investigate if there were local outbreaks that could be related to a domestic intensification of insecticide use and, potentially, to an increase in* A. aegypti* resistance to these compounds. The period chosen ranged from 2008, two years before the collection of the first* A. aegypti* samples here evaluated, up to 2012. Incidence values for municipalities and the corresponding states are also presented in Table S1 of the Supplementary Material available online at http://dx.doi.org/10.1155/2016/8603263.

According to the Brazilian MoH, dengue incidence rates higher than 300 cases/100,000 inhabitants are already indicative of an epidemic situation [50]. In almost all municipalities, at least once, the dengue incidence of notified cases was compatible with this scenario. When the whole 2008–2012 period was considered, only Marabá, PA, was the exception. However, the localities of Castanhal, PA, and Caicó, RN, only presented high dengue incidence at 2012, after collection of vector samples was made (compare Figures 2 and 3). In some situations this “epidemic status” persisted throughout the whole evaluated period; this was the case of Pacaraima, RR, and of the GO adjacent municipalities Goiânia and Aparecida de Goiânia. The number of registers well above the threshold value of 300 cases per 100,000 inhabitants also attracted attention. For instance, reported incidence equivalent to at least 1% (1,000 cases/100,000 inhabitants) was found during 2009 and 2010 in half of the evaluated municipalities. Notably, the dengue incidence of Aparecida de Goiânia remained above 1% during the whole evaluated period. In the adjacent locality, Goiânia, those high dengue rates were registered during three years between 2008 and 2012. One should be aware that in general dengue epidemic periods are related to a significant increase in the domestic use of insecticides against adult mosquitoes (see Section 4).

### 3.3. Bioassays with Larvae

Table 1 summarizes the results of quantitative bioassays performed with the two main larvicides recently employed against* A. aegypti* in Brazil, the OP temephos and the CSI diflubenzuron. Table S2 shows details of these assays, such as effective concentrations and confidence intervals. Data are organized by year and then by geographic region.

All the populations evaluated were considered resistant to temephos. The higher resistance values were obtained in 2012. Nevertheless, since there were no municipalities examined in consecutive years, it is not possible to claim that temephos resistance is increasing in the country, based on the data presented here. In general, temephos resistance was higher at the central-west region: six out of seven municipalities presented RR_95_ above 10. São Miguel do Araguaia, GO, exhibited the higher temephos RR_95_ value, above 27. In contrast, Pacaraima, the municipality with the lower resistance level to the OP, is located at RR, a state where temephos supply started later than in the other states (Figure 2). In comparison with Rockefeller, a general higher heterogeneity of field populations was detected, as judged by their low slope values.

In contrast to the results obtained for temephos, all populations analyzed were susceptible to diflubenzuron (RR_95_ < 3.0). This was true even for mosquito populations bearing high temephos resistance rates, suggesting absence of cross resistance between these compounds in the localities examined. In contrast to the results obtained with temephos, field populations seemed more homogeneous than Rockefeller strain regarding diflubenzuron resistance profiles.

### 3.4. Bioassays and Molecular Assays with Adults

Resistance rates resulting from bioassays with the adulticide PY deltamethrin are depicted in Table 2, together with the Na_V_ allelic frequencies, where R1 and R2 are the kdr alleles related to PY target site resistance. Additional details of the bioassays are presented in Table S3 that also includes the kdr allelic frequencies for each position (1016 and 1534) separately [20].

Very high deltamethrin resistance levels were found for all populations; RR_95_ was always above 10.0. Caicó, RN, at the northeast region, and Castanhal, PA, in the north region, exhibited the lowest RR_95_, respectively, 13.1 and 14.9. Accordingly, these municipalities presented the lower frequencies of R1 and R2 kdr alleles. In all other municipalities values remained above 45.0. In two localities, Marabá, PA, and Cachoeiro de Itapemirim, ES, RR_95_ was higher than 70.0. In this latter locality, due to the high resistance level detected, there was lack of enough specimens to reach LC_95_ (note that in Tables 2 and S3 the higher value shown for Cachoeiro de Itapemirim corresponds to LC_80_). The supply of pyrethroids for the bulk of the states evaluated here was continuous since at least 2003 (Figure 2). Comparison of the slope values obtained for deltamethrin assays shows that, in general, field populations evaluated demonstrate higher heterogeneity than the Rockefeller strain.

### 3.5. Biochemical Assays

Tables 3 and 4 present the results of biochemical assays for, respectively, larval and adult stages. In both tables, data are organized by decreasing RR_95_ order for the neurotoxic insecticides temephos (larvae) and deltamethrin (adults). Additional details of these assays are shown in Tables S4 and S5.

According to the WHO criterion, measurements of inhibition of AChE activity, the OP target site, point to unaltered activity for all populations and development stages (data not shown). This was verified because the carbamate propoxur induced more than 70% of AChE activity inhibition in all cases [47]. In addition, quantification of total AChE activity [45, 46] revealed values compatible with susceptibility, in all cases, with exception of adults from one population (São Simão, GO, Table 4).

For all the remaining enzymes, changes were detected for both stages. In the larval stage, major changes were noted in the activities of GST, *β*-EST, and *ρ*NPA-EST, while moderate increases were noted for MFO and *α*-EST. The intensity of enzymatic alterations appeared to be higher at the adult stage, mainly when MFO, GST, and *α*-EST were considered. In general, populations with a higher RR also tended to exhibit a higher increase in detoxifying enzymes activity, taking into account both the number of altered classes and the intensity of activity increment.

## 4. Discussion

The use of insecticides in the control of the dengue vector in Brazil has been broad and continuous, a procedure that favored the selection of resistant specimens over the years. In order to assist in the rational use of pesticides, in 2006, the Brazilian Dengue Control Program adopted a functional criterion for the evaluation of the temephos status of* A. aegypti* populations. This criterion, also employed here to classify both deltamethrin and diflubenzuron resistance status, considers that populations with RR_95_ above 3.0 are resistant. This is the cutoff to conduct insecticide substitution in the field, and the adoption of this parameter took into account Brazilian operational aspects of insecticide management, like the period of time necessary for the effective implementation of the insecticide substitution in all affected localities. This strategy aimed to preserve the insecticides in the field [23].

Functional validation of this criterion has been previously obtained through simulated field assays with temephos and, more recently, with pyrethroids [23, 51]. Resistance to diflubenzuron was not established in the country and therefore a functional criterion has not yet been defined for this IGR by PNCD. However, Fontoura et al. [42], using simulated assays, did not find impairment of the efficacy of another CSI, novaluron, in* A. aegypti* populations bearing RR_90_ < 2.0.

The use of OP pesticides in Brazil for* A. aegypti* control dates back to the 1960s, and it was intensified since 1986, when the DENV-1 virus was introduced in the country [52, 53]. As a result, resistance to temephos has been reported in Brazilian populations of* A. aegypti* collected from 1998 on [23, 27, 28]. Resistance to temephos spread around the country so intensely that, since 2009, PNCD does not recommend the use of this OP as the larvicide of choice [54]. Accordingly, all populations here evaluated between 2010 and 2012 were resistant to temephos.

Investigation of putative resistance mechanisms present in* A. aegypti* larvae excluded the participation of acetylcholinesterase, the OP target site. Regarding metabolic resistance, MFO enzymes are strongly associated with insecticide resistance in several* A. aegypti* populations around the world [25, 26, 55]. We found only discrete alterations in this class of enzymes in larvae from Brazilian* A. aegypti* populations, while adult specimens exhibited levels of MFO alteration equivalent to EST and GST enzymes. In 2007,* A. aegypti* adult EST activities were associated with resistance to both OP and PY in Brazil [23]. Connections between OP resistance and significant alterations of EST as well as association between PY resistance and both MFO and GST elevated activity rates were also reported in other countries [25, 26, 55].

In general, higher RR levels against OP and PY neurotoxic insecticides correlated to increased metabolic alterations in terms of both number of enzymes affected and intensity of activity enhancement [30, 55, 56]. Usually detoxifying enzymes that trigger metabolic resistance participate in the general insect metabolism. These are somewhat generic molecules, with a variable affinity for a number of insecticides or other xenobiotics. Although resistance to the IGR diflubenzuron has not yet been detected in the country, the development of metabolic resistance against these compounds is a potential mechanism that should be monitored.

For some localities here depicted, previous evaluations of the temephos susceptibility status are available. In these cases, the resistance dynamics profiles were compared to the insecticide distribution performed by the MoH to each state (Figure 2). Increase in the temephos resistance status was noted whenever the application of this OP persisted. Examples are the central-west municipalities of Goiânia (GOI) and Aparecida de Goiânia (APG), at GO state: between 2003 and 2011, GOI RR_95_ increased more than twice, from 3.3 to 8.6 (Table 1, [23]). In APG the temephos RR_95_ also increased significantly between 2006 (11.2) and 2012 (16.6) (Table 1, [57]). In contrast, only a low decrease in the temephos resistance status was noted following its interruption. This was the case of Caicó (CAC), at RN state, where no temephos was provided since 2004 (Figure 2). At that year, CAC temephos RR_95_ was 12.5; six years later this value dropped only slightly, to 9.6 (Table 1, [23]).

One major consequence of the high and disseminated Brazilian* A. aegypti* temephos resistance status was the inclusion of the CSI diflubenzuron in the chemical control of larvae since 2009. Bioassays of mosquito samples obtained between 2010 and 2012 confirmed the susceptible status of all evaluated populations to this product. Diflubenzuron resistance ratios below 3.0 were also found for field* A. aegypti* populations from Cabo Verde, Malaysia, and Martinique [58–60]. Together, these data point that the use of this class of insecticides in the control of larvae of the dengue vector is still viable.

The bulk of results obtained by the* A. aegypti* insecticide resistance monitoring Brazilian network guided the option to rotate products against larvae in the country. Insect Growth Regulators were adopted. Due to operational issues, the maximum period of four years was fixed for alternation of products [61]. The aim of this resistance management strategy was to preserve the few available larvicides. In this regard, it is ought to mention that Brazil only employs larvicides recommended by WHOPES for use in drinking water [14]. Currently, the IGR used against* A. aegypti* larvae is the juvenile hormone analogue (JHA) pyriproxyfen [62].

Some decades were necessary until the spread of resistance to the OP temephos in Brazil could compromise its use in the control of* A. aegypti*. In contrast, in the case of pyrethroids, introduced in the whole country in 2000 by PNCD, only a few years were enough to the achievement of extremely high resistance levels [23, 31, 32]. If, on the one hand, chemical control of* A. aegypti* adults is recommended by the MoH only to block outbreaks or on the imminence of a dengue epidemic [9], on the other hand, unlike temephos, PY insecticides are available in the retail market. The domestic use of pyrethroids is intensified at every dengue epidemic period and certainly contributes significantly to the rapid dissemination of resistance [33]. Accordingly,* A. aegypti* deltamethrin resistance levels doubled between 2009 and 2011 at Cachoeiro de Itapemirim and Goiânia; in the same period, an eightfold increase was observed for this parameter at Marabá (Table 2; Bellinato D, personal communication). Cachoeiro de Itapemirim and Goiânia faced dengue outbreaks in this interval, corroborating the hypothesis that the intensification of the domestic use of PY collaborated in the resistance increase. However no dengue outbreak was noted at Marabá in this period. Marabá is located in the Amazon region, where almost all Brazilian malaria cases are reported. Control of* Anopheles* malaria vectors also employs PY and this could explain the increased resistance observed. In contrast, the lowest PY resistance levels were found for Castanhal at 2010 and Caicó at 2011, two municipalities that had not experienced dengue epidemics since 2008.

To date, the PY resistance ratios found here are among the highest ones reported in the country. In spite of that, heterogeneity levels exhibited by those vector populations suggest that the insecticide resistance character is still not irreversibly fixed. In relation to the PY target site resistance, the voltage gated sodium channel, this heterogeneity confirms previous observations, reporting the presence of the Na_V_ susceptible allele (S) with allelic frequencies between 0.0 and 0.92 (Table 2). Regarding metabolic resistance of adult mosquitoes, as for temephos resistance and larvae biochemical profile (mentioned above), a general positive correlation between the resistance level and the magnitude of altered enzymes was also found. It should be noted that, in this case, except for Caicó, all populations exhibited PY resistance levels above 45.0. Hence, while Caicó detoxifying enzymes effects were restricted to esterases, in the remaining populations, GST or MFO activities (and both enzymes in some cases) were significantly enhanced. In addition, GST, MFO, and EST have already been correlated with PY resistance [5, 23, 44, 63].

Due to the spread of high pyrethroid resistance levels throughout the country, since 2009 PY is being gradually replaced by the OP malathion for the control of* A. aegypti* adults, employed in residual applications and UBV [16, 54, 64].

Currently, chemical control is still largely applied in public health, favoring the insecticide resistance dissemination of vector populations. Accordingly, we detected high resistance levels against the OP temephos and the PY deltamethrin, insecticides long used in the country. In some municipalities, comparison with the incidence of dengue outbreaks suggested significant participation of the domestic use of PY insecticides in the rapid resistance increase. The introduction of IGRs is recent in Brazil, and all* A. aegypti* populations here evaluated were susceptible to the CSI diflubenzuron. Together our results indicate the potential of this IGR against the vector but also point to the need for rational use of chemical tools. In this sense, the adoption of rotation of compounds with different mechanisms of action is a positive step. It still remains, however, to invest in awareness campaigns, directed to both managers and the general society, regarding the importance of the mechanical control of vectors as a priority. Spreading the concept that chemical control is a complementary antivector strategy is the best way to preserve insecticides.

## Supplementary Material

Table S1 corresponds to data used to construct Figure 3. It presents the incidence of dengue cases, between 2008 and 2012, in the evaluated municipalities. Tables S2 and S3 contain additional data obtained for respectively larvae and adults (with bioassays and molecular assays): Table S2 complements Table 1 (temephos and diflubenzuron results) and Table S3 complements Table 2 (deltamethrin bioassays and pyrethroid target site data). Regarding bioassays, effective doses and confidentiality intervals are shown. For molecular assays, the frequencies of individual substitutions, at 1016 and 1534 positions, are depicted. Tables S4 and S5 give details of the biochemical assays, such as number of individual specimens tested in each case, the median values of all enzyme activities for the evaluated populations and the Rockefeller 99 percentile used to classify activities. Tables S4 and S5 also repeat the rate of specimens with activity higher than the Rockefeller 99 percentile, the parameter used to classify the populations (that is shown here in colors).

## Figures and Tables

**Table 1:** Resistance status of several Brazilian municipalities to the larvicides temephos (OP) and diflubenzuron (CSI).

Year	Region	State	Municipality/strain	Generation	Temephos			Generation	Diflubenzuron		
					RR_50_	RR_95_	Slope		RR_50_	RR_95_	Slope
2010	NE	RN	Rockefeller		1.0	1.0	6.20		1.0	1.0^*∗*^	4.85
			Caicó	F2	8.4	**9.6**	5.07	F2	2.2	*1.7* ^*∗*^	6.56

2011	N	RR	Rockefeller		1.0	1.0	5.03		1.0	1.0	4.16
			Pacaraima	F2	4.3	**4.0**	5.70	F2	1.7	*1.5*	5.05
		PA	Castanhal	F2	8.2	**11.2**	3.53	F2	1.4	*1.2*	4.75
			Marabá	F2	8.0	**10.3**	3.76	F3	1.8	*1.6*	4.91
	SE	ES	Cachoeiro de Itapemirim	F1	18.4	**17.1**	5.57	F2	1.9	*1.6*	5.03
	CW	GO	Campos Belos	F2	9.1	**12.0**	3.68	F2	1.7	*1.6*	4.38
			Goiânia	F2	7.9	**8.6**	4.56	F2	1.6	*1.8*	3.63
			Rio Verde	F2	11.5	**14.8**	3.77	F2	2.0	*1.6*	5.32
			São Simão	F2	12.1	**14.8**	3.98	F2	2.0	*2.3*	3.57
			Uruaçu	F2	10.5	**12.5**	4.08	F2	1.9	*1.5*	5.78

2012	CW	GO	Aparecida de Goiânia	F1	17.9	**16.6**	5.59	F2	1.1	*2.1*	2.43
			São Miguel do Araguaia	F1	21.1	**27.1**	3.77	F1	1.6	*1.7*	3.82

RR_50_ and RR_95_: resistance ratios; profiles corresponding to RR_95_ below or above 3.0 (italic font or bold font numbers) were classified as susceptible or resistant, respectively.

^*∗*^RR_80_ is informed. See Table S2 for additional details.

**Table 2:** Resistance status of several Brazilian municipalities to the pyrethroid deltamethrin and allelic frequencies of the major kdr mutations found in the country.

Year	Region	State	Municipality/strain	Generation	RR_50_	RR_95_	Slope	Na_V_ allelic frequencies		
								S	R1	R2
2010	NE	RN	Rockefeller		1.0	1.0	2.96			
			Caicó	F2	6.0	**13.1**	2.51	0.917	0.067	0.017

2011	N	RR	Rockefeller		1.0	1.0	4.55			
			Pacaraima	F2	33.2	**60.3**	2.65	0.000	0.600	0.400
		PA	Castanhal	F2	9.9	**14.9**	3.05	0.667	0.300	0.033
			Marabá	F2	47.4	**70.7**	3.07	0.690	0.310	0.000
	SE	ES	Cachoeiro de Itapemirim	F1	49.0	78.6^*∗*^	2.16	0.103	0.224	0.672
	CW	GO	Campos Belos	F2	25.3	**52.3**	2.43	0.310	0.086	0.603
			Goiânia	F2	47.6	**46.5**	4.68			
			Rio Verde	F2	32.5	**56.2**	2.74	0.241	0.207	0.552
			São Simão	F2	30.4	**51.6**	2.78	0.083	0.383	0.533
			Uruaçu	F2	38.6	**51.6**	3.37	0.450	0.117	0.433

2012	CW	GO	Aparecida de Goiânia	F1	33.0	**57.2**	2.74	0.207	0.362	0.463
			São Miguel do Araguaia	F1	39.6	**49.4**	3.59	0.414	0.293	0.293

RR_50_ and RR_95_: resistance ratios; profiles corresponding to RR_95_ above 3.0 (bold font numbers) were classified as resistant.

^*∗*^RR_80_ is informed. See Table S3 for additional details.

Kdr allelic frequencies S, R1, and R2 refer to the positions 1016 and 1534 of the gene coding for the voltage gated sodium channel (Na_V_) as follows: S (susceptible) = 1016 Val^+^/1534 Phe^+^; R1 (single mutant) = 1016 Val^+^/1534 Cys^kdr^; and R2 (double mutant) = 1016 Ile^kdr^/1534 Cys^kdr^. Kdr data have been originally published by Linss et al. 2014 [20].

**Table 3:** Quantification of the enzymatic activity of *A. aegypti* larvae from different Brazilian municipalities. Numbers refer to the rate of specimens with activity higher than the 99 percentile of Rockefeller (%>p99). Municipalities are arranged in descending order of temephos resistance (RR_95_ OP).

Year	Region	State	Municipality/strain	RR_95_ OP	ACE	MFO	GST	A-EST	B-EST	PNPA-EST
2012	CO	GO	São Miguel do Araguaia	**27.1**	0	0	$\underline{\text{20}}$	$\underline{\text{28}}$	$\underline{\text{21}}$	***59***
2012	CO	GO	Aparecida de Goiânia	**16.6**	5	$\underline{\text{31}}$	***85***	8	***54***	***63***
2011	CO	GO	Rio Verde	**14.8**	1	0	$\underline{\text{40}}$	$\underline{\text{19}}$	$\underline{\text{38}}$	8
2011	CO	GO	São Simão	**14.8**	1	4	$\underline{\text{40}}$	$\underline{\text{35}}$	$\underline{\text{33}}$	$\underline{\text{32}}$
2011	CO	GO	Campos Belos	**12.0**	1	15	9	1	5	0
2011	N	PA	Castanhal	**11.2**	0	$\underline{\text{48}}$	$\underline{\text{34}}$	3	$\underline{\text{29}}$	***75***
2010	NE	RN	Caicó	**9.6**	0	4	$\underline{\text{19}}$	0	0	0
2011	CO	GO	Goiânia	**8.6**	3	6	$\underline{\text{49}}$	5	$\underline{\text{34}}$	$\underline{\text{36}}$
2011	N	RR	Pacaraima	**4.0**	6	1	$\underline{\text{21}}$	0	14	3

Activities were classified as normal (regular font), altered (italic and underlined font) or highly altered (italic and bold) if these values ranged respectively below 15%, between 15 and 50% or above 50%.

**Table 4:** Quantification of the enzymatic activity of *A. aegypti* adults from different Brazilian municipalities. Numbers refer to the rate of specimens with activity higher than the 99 percentile of Rockefeller (%>p99). Municipalities are arranged in descending order of deltamethrin resistance (RR_95_ PI).

Year	Region	State	Municipality/strain	RR_95_ PI	ACE	MFO	GST	A-EST	B-EST	PNPA-EST
2011	SE	ES	Cachoeiro de Itapemirim	78.6^**∗**^	0	***80***	***98***	***67***	3	***70***
2011	N	RR	Pacaraima	**60.3**	0	$\underline{\text{17}}$	$\underline{\text{40}}$	$\underline{\text{48}}$	13	2
2012	CO	GO	Aparecida de Goiânia	**57.2**	1	***57***	***94***	***70***	10	***73***
2011	CO	GO	Rio Verde	**56.2**	13	***74***	4	***81***	8	13
2011	CO	GO	Campos Belos	**52.3**	0	8	***65***	14	1	6
2011	CO	GO	São Simão	**51.6**	$\underline{\text{21}}$	9	***59***	***58***	14	15
2012	CO	GO	São Miguel do Araguaia	**49.4**	4	***97***	8	$\underline{\text{46}}$	0	8
2011	CO	GO	Goiânia	**46.5**	0	***99***	***78***	***55***	5	$\underline{\text{38}}$
2010	NE	RN	Caicó	**13.1**	3	0	11	***63***	$\underline{\text{22}}$	6

Activities were classified as normal (regular font), altered (italic and underlined font) or highly altered (italic and bold) if these values ranged respectively below 15%, between 15 and 50% or above 50%. ^*∗*^RR_80_ is informed.
