# Supplementary material for: Resistance Status to the Insecticides Temephos, Deltamethrin, and Diflubenzuron in Brazilian Aedes aegypti Populations
Source: Biomed Res Int. 2016 Jun 21;2016:8603263. doi: 10.1155/2016/8603263 (PMC4932163; doi:10.1155/2016/8603263)
Supplement: Supplementary file 1 — Table S1 corresponds to data used to construct Figure 3. It presents the incidence of dengue cases, between 2008 and 2012, in the evaluated municipalities. Tables S2 and S3 contain additional data obtained for respectively larvae and adults (with bioassays and molecular assays): Table S2 complements Table 1 (temephos and diflubenzuron results) and Table S3 complements Table 2 (deltamethrin bioassays and pyrethroid target site data). Regarding bioassays, effective doses and confidentiality intervals are shown. For molecular assays, the frequencies of individual substitutions, at 1016 and 1534 positions, are depicted. Tables S4 and S5 give details of the biochemical assays, such as number of individual specimens tested in each case, the median values of all enzyme activities for the evaluated populations and the Rockefeller 99 percentile used to classify activities. Tables S4 and S5 also repeat the rate of specimens with activity higher than the Rockefeller 99 percentile, the parameter used to classify the populations (that is shown here in colors). [file 8603263.f1.pdf]

Table S1: incidence of reported dengue cases in the municipalities evaluated from 2008 to 2012 and in the corresponding States (in grey). Values refer to the rate of notified cases per 100.000 inhabitants.

Region	State	Locality	2008	2009	2010	2011	2012
N	RR		**1,194**	**756**	**1,685**	**322**	**399**
N	RR	Pacaraima	**700**	**374**	**1,074**	**460**	**939**
N	PA		211	103	205	254	208
N	PA	Castanhal	131	95	131	76	**1,588**
N	PA	Marabá	252	106	129	214	141
NE	RN		**918**	81	248	**731**	**892**
NE	RN	Caicó	217	6	108	234	**506**
SE	ES		**756**	**767**	**742**	**1,148**	**334**
SE	ES	Cachoeiro de Itapemirim	**3,271**	7	**798**	**775**	**320**
CO	GO		**609**	**646**	**1,700**	**566**	**398**
CO	GO	Aparecida de Goiânia	**1,293**	**1,821**	**2,138**	**1,169**	**1,536**
CO	GO	Campos Belos	217	**1,505**	4,356	92	5
CO	GO	Goiânia	**1,529**	**1,841**	**3,217**	**470**	**781**
CO	GO	Rio Verde	**763**	184	**1,721**	249	**321**
CO	GO	São Miguel do Araguaia	**332**	**1,414**	**574**	117	94
CO	GO	São Simão	12	**1,375**	**2,382**	99	59
CO	GO	Uruaçu	62	**1,595**	141	111	76

Available at [http://www.censo2010.ibge.gov.br/sinopse/index.php?uf=14&dados=21], acessed at 3rd Aug 2015.

Bold: values above 300, a high incidence rate according to the Brazilian Ministry of Health

Table S2: temephos lethal concentrations (LC) and diflubenzuron adult emergence inhibition doses (EI) of several Brazilian municipalities.

year	Region	State	Municipality/strain	temephos				diflubenzuron			
				LC_{50} (µg/L)	LC_{95}(µg/L)	range		EI_{50} (µg/L)	EI_{95}(µg/L)	range	
						LC_{50} (µg/L)	LC_{95} (µg/L)			EI_{50} (µg/L)	EI_{95} (µg/L)
2010	NE	RN	Rockefeller	2.89	5.33	2.80 < LC < 2.98	5.17 < LC < 5.50	1.101	1.900*	1.047 < LC < 1.176	1.805 < LC < 1.999*
			Caicó	24.31	51.31	23.68 < LC < 24.95	48.94 < LC < 53.80	2.444	3.283*	2.362 < LC < 2.528	3.119 < LC < 3.456*
2011			Rockefeller	3.56	7.56	3.31 < LC < 3.82	6.75 < LC < 8.46	0.970	2.410	0.918 < LC < 1.025	2.256 < LC < 2.574
	N	RR	Pacaraima	15.38	29.91	13.49 < LC < 17.54	25.42 < LC < 35.19	1.695	3.591	1.608 < LC < 1.788	3.307 < LC < 3.898
		PA	Castanhal	29.03	85.01	25.18 < LC < 33.47	67.00 < LC < 107.85	1.312	2.912	1.239 < LC < 1.389	2.662 < LC < 3.184
			Marabá	28.45	77.80	25.62 < LC < 31.59	66.28 < LC < 91.31	1.791	3.874	1.708 < LC < 1.878	3.561 < LC < 4.213
	SE	ES	Cachoeiro de Itapemirim	65.49	129.35	61.86 < LC < 69.33	118.68 < LC < 140.97	1.846	3.921	1.757 < LC < 1.940	3.639 < LC < 4.224
	CW	GO	Campos Belos	32.47	90.87	30.12 < LC < 35.00	78.91 < LC < 104.65	1.654	3.924	1.568 < LC < 1.744	3.530 < LC < 4.362
			Goiânia	28.25	64.83	25.83 < LC < 30.90	55.50 < LC < 75.73	1.509	4.285	1.414 < LC < 1.610	3.754 < LC < 4.891
			Rio Verde	40.99	112.06	37.04 < LC < 45.36	96.92 < LC < 129.57	1.914	3.902	1.830 < LC < 2.002	3.573 < LC < 4.262
			São Simão	43.13	111.77	37.50 < LC < 49.68	92.73 < LC < 134.71	1.953	5.637	1.851 < LC < 2.061	5.019 < LC < 6.331
			Uruaçu	37.37	94.53	32.88 < LC < 42.46	79.93 < LC < 111.81	1.877	3.615	1.799 < LC < 1.958	3.335 < LC < 3.919
2012	CW	GO	Aparecida de Goiânia	63.68	125.36	60.03 < LC < 67.54	115.45 < LC < 136.12	1.069	5.078	0.967 < LC < 1.181	4.201 < LC < 6.137
			São Miguel do Araguaia	75.05	205.21	68.19 < LC < 82.59	175.02 < LC < 240.61	1.508	4.067	1.417 < LC < 1.605	3.597 < LC < 4.599

* EI_{80} is informed.

Table S3: deltamethrin lethal concentrations (LC) and *kdr* frequencies of several Brazilian municipalities. *Kdr* data source: Linss *et al* 2014.

year	Region	State	Municipality/strain	Deltamethrin LC_{50} (mg/L)	LC_{80}(mg/L)	LC_{95}(mg/L)	range LC_{50} (mg/L)	range LC_{80} (mg/L)	range LC_{95} (mg/L)	generation	sample size	1016Ile frequency	1534Cys frequency	Na_v allelic frequencies S	R1	R2
			Rockefeller	0.70		1.45	0.70 < LC < 0.81		2.36 < LC < 3.13							
2010	NE	RN	Caicó	4.22		19.04	3.62 < LC < 4.92		15.32 < LC < 23.67	F1	30	0.017	0.084	0.917	0.067	0.017
			Rockefeller	0.92	1.41	2.12	0.84 < LC < 1.01	1.30 < LC < 1.54	1.87 < LC < 2.41							
		RR	Pacaraima	30.61		127.89	25.96 < LC < 36.10		100.19 < LC < 163.25	F0	30	0.400	1.000	0.000	0.600	0.400
	N	PA	Castanhal	9.12		31.52	8.03 < LC < 10.35		26.97 < LC < 36.86	F0	30	0.033	0.333	0.667	0.300	0.033
			Marabá	43.72		149.97	38.73 < LC < 49.36		115.85 < LC < 194.16	F0	29	0.000	0.310	0.690	0.310	0.000
	SE	ES	Cachoeiro de Itapemirim	45.19	110.98		41.61 < LC < 49.10	96.72 < LC < 127.37		F0	29	0.672	0.896	0.103	0.224	0.672
2011			Campos Belos	23.37		110.84	17.96 < LC < 30.42		82.69 < LC < 148.58	F0	29	0.603	0.689	0.310	0.086	0.603
			Goiânia	43.88		98.57	41.23 < LC < 46.70		89.75 < LC < 108.27							
	CW	GO	Rio Verde	30.01		119.29	25.35 < LC < 35.51		94.31 < LC < 150.89	F0	29	0.552	0.759	0.241	0.207	0.552
			São Simão	28.08		109.52	25.53 < LC < 33.52		87.71 < LC < 136.77		30	0.533	0.916	0.083	0.383	0.533
			Uruaçu	35.64		109.54	32.36 < LC < 39.24		97.18 < LC < 123.49	F0	30	0.433	0.550	0.450	0.117	0.433
2012	CW	GO	Aparecida de Goiânia	30.48		121.39	27.17 < LC < 34.19		103.05 < LC < 143.00	F0	29	0.4631	0.825	0.207	0.362	0.463
			São Miguel do Araguaia	36.51		104.87	34.26 < LC < 38.90		95.79 < LC < 114.81	F0	29	0.293	0.586	0.414	0.293	0.293

* RR_{80} is informed.

Alleles S, R1 and R2 refer to the positions 1016 and 1534 of the gene coding for the voltage gated sodium channel (Na_V) as follows: S = 1016 Val+ / 1534 Phe+ ; R1 = 1016 Val+ / 1534 Cyskdr ; R2 = 1016 Ilekdr / 1534 Cyskdr.

Table S4: details of the of the enzimatic activity quantification in Brazilian *Aedes aegypti larvae* populations. Colors as in Table 3 and 4.

year	Region	State	Municipility/strain	generation	ACE (Abs/hour/ptn mg)				MFO (nmoles cit/ptn mg)				GST (mmol/min/ptn mg)				α-Est (nmol/ptn mg/min)				β-Est (nmol/ptn mg/min)				ρ npa-Est (D Abs/min/ptn mg)			
					n	med	p99 Rock	% > p99 Rock	n	med	p99 Rock	% > p99 Rock	n	med	p99 Rock	% > p99 Rock	n	med	p99 Rock	% > p99 Rock	n	med	p99 Rock	% > p99 Rock	n	med	p99 Rock	% > p99 Rock
2010			Rockefeller		256	0.04	0.14		268	40.88	78.41		265	0.38	1.10		268	17.06	62.02		268	29.65	82.75		249	3.02	5.46	
	NE	RN	Caicó	F2	83	0.03		0	83	38.50		4	83	0.78		19	55	22.41		0	55	30.05		0	54	2.62		0
			Rockefeller		179	0.05	0.09		172	35.57	67.48		153	0.47	0.94		178	13.67	22.24		163	19.56	31.19		173	2.47	4.19	
	N	PA	Pacaraima	F2	80	0.06		6	79	34.56		1	78	0.57		21	80	12.09		0	80	21.66		14	77	1.99		3
			Castanhal	F2	80	0.04		0	80	65.12		48	80	0.76		34	80	15.33		3	80	27.69		29	80	5.29		75
2011			Campos Belos	F2	79	0.05		1	79	26.32		15	77	0.50		9	79	13.73		1	79	22.46		5	72	1.86		0
	CO	GO	Goiânia	F2	80	0.06		3	80	40.18		6	80	0.93		49	80	16.39		5	80	28.38		34	80	3.75		36
			Rio Verde	F2	81	0.05		1	81	25.63		0	81	0.75		40	81	18.68		19	81	29.54		38	78	2.41		8
			São Simão	F2	78	0.06		1	80	36.84		4	80	0.87		40	79	18.98		35	80	27.41		33	77	3.47		32
2012	CO	GO	Aparecida de Goiânia	F1	80	0.06		5	80	53.97		31	80	1.28		85	80	16.05		8	80	31.91		54	80	4.40		63
			São Miguel do Araguaia	F1	80	0.04		0	80	30.76		0	80	0.73		20	80	19.41		28	80	26.44		21	80	4.58		59

The Rockefeller values used to callibrate each group of assays are shown above the municipalities. (n) Number of specimens evaluated. (med) Median of each enzimatic activity. (p99 Rock) 99 percentil for Rockefeller strain. (%>p99Rock) Rate of the population with activity higher than the Rockefeller 99 percentil.

Table S5: details of the of the enzimatic activity quantification in Brazilian Aedes aegypti adult populations. Colors as in Table 3 and 4.

year	Region	State	Municipility/strain	generation	ACE (Abs/hour/ptn mg)				MFO (nmoles cit/ptn mg)				GST (mmol/min/ptn mg)				α-Est (nmol/ptn mg/min)				β-Est (nmol/ptn mg/min)				ρ npa-Est (D Abs/min/ptn mg)			
					n	med	p99 Rock	% > p99 Rock	n[1]	med[2]	p99 Rock[3]	% > p99 Rock[4]	n	med	p99 Rock	% > p99 Rock	n	med	p99 Rock	% > p99 Rock	n	med	p99 Rock	% > p99 Rock	n	med	p99 Rock	% > p99 Rock
2010			Rockefeller		132	0.08	0.12		122	23.89	44.02		124	0.95	1.45		129	4.77	6.32		131	5.96	9.00		98	3.24	6.42	
	NE	RN	Caicó	F2	71	0.08		3	96	26.41		0	93	1.13		11	100	6.62		63	109	5.70		22	97	3.30		6
			Rockefeller		155	0.12	0.22		137	28.05	44.97		100	0.66	1.07		154	5.49	7.58		153	6.33	10.77		83	3.20	6.39	
	N	RR	Pacaraima	F2	80	0.17		0	77	38.59		17	60	1.00		40	80	7.52		48	80	8.61		13	65	4.26		2
	SE	ES	Cachoeiro de Itapemirim		80	0.12		0	79	54.8		80	80	1.60		98	79	8.12		67	80	7.43		3	73	7.71		70
2011			Campos Belos	F2	80	0.15		0	80	28.64		8	78	1.22		65	80	6.45		14	80	6.25		1	64	4.58		6
	CO	GO	Goiânia	F2	80	0.16		0	78	70.3		99	79	1.25		78	80	7.90		55	80	7.72		5	78	6.23		38
			Rio Verde	F2	80	0.18		13	78	48.27		74	45	0.64		4	80	8.98		81	79	7.85		8	75	3.76		13
			São Simão	F2	80	0.18		21	78	30.98		9	54	1.12		59	80	8.03		58	80	6.98		14	67	5.04		15
2012	CO	GO	Aparecida de Goiânia	F1	80	0.13		1	77	63.42		57	68	1.58		94	80	8.54		70	80	8.12		10	79	7.40		73
			São Miguel do Araguaia	F1	80	0.18		4	78	62.41		97	52	0.78		8	80	7.50		46	79	6.55		0	79	4.80		8

The Rockefeller values used to callibrate each group of assays are shown above the municipalities. (n) Number of specimens evaluated. (med) Median of each enzimatic activity. (p99 Rock) 99 percentil for Rockefeller strain. (%>p99Rock) Rate of the population with activity higher than the Rockefeller 99 percentil.
